# The association of female reproductive factors with history of cardiovascular disease: a large cross-sectional study

**DOI:** 10.1186/s12889-024-19130-4

**Published:** 2024-06-17

**Authors:** Tiehan Chen, Jingwen Wu, Qinyuan Pan, Mingmei Dong

**Affiliations:** 1 Department of Internal Medicine, Lianyungang Maternal and Child Health Hospital, Lianyungang, Jiangsu 222000 China; 2 Department of Cardiology, Lianyungang First People’s Hospital, Lianyungang, Jiangsu 222000 China; 3 Department of Intensive Care Unit, Lianyungang First People’s Hospital, Lianyungang, Jiangsu 222000 China

**Keywords:** Age at first birth, Age at last birth, Cardiovascular disease, Number of pregnancies, Number of live births

## Abstract

**Background:**

This study aimed to explore the association of female reproductive factors (age at first birth (AFB), age at last birth (ALB), number of pregnancies, and live births) with history of cardiovascular disease (CVD).

**Methods:**

A total of 15,715 women aged 20 years or over from the National Health and Nutrition Examination Surveys from 1999 to 2018 were included in our analysis. Weighted multivariable logistic regression analysis and restricted cubic spline (RCS) model were used to evaluate the association of AFB and ALB with history of CVD in women. Additionally, the relationship between the number of pregnancies, and live births and history of CVD was also explored.

**Results:**

After adjusting for potential confounding factors, the RCS plot showed a U-curve relationship between AFB, ALB and history of CVD. Among them, AFB was associated with congestive heart failure (CHF), heart attack, and stroke in a U-shaped curve. Additionally, this U-shaped correlation also exists between ALB and CHF and stroke. However, the number of pregnancies and live births was liner positive associated with history of CVD, including coronary heart disease, CHF, angina pectoris, heart attack, and stroke.

**Conclusions:**

Women with younger or later AFB and ALB have higher odds of CVD in later life. Further study is warranted to verify the underlying mechanisms of this association.

**Supplementary Information:**

The online version contains supplementary material available at 10.1186/s12889-024-19130-4.

## Introduction

Cardiovascular disease (CVD) is the leading cause of death worldwide, contributing to about 17.9 million deaths in 2016, or 31% of all global deaths [1]. Additionally, CVD is the main cause of mortality in the United States (U.S.), accounting for approximately one death out of every seven [2]. Age, hypertension, elevated blood glucose, dyslipidemia, overweight and obesity, etc. are among the traditional risk factors for CVD [3].

Women and men may have different risk factors for CVD. Until menopause, women appear to be at lower risk of CVD than men [4]. However, women’s CVD risk increases after menopause and becomes similar to that of men [5]. The estrogen level in women changes dramatically with age and drops rapidly after menopause, which profoundly affects their health [6]. El Khoudary SR et al. has found that postmenopausal women’s decreased estrogen levels are believed to contribute to the rise in CVD in postmenopausal women [7]. Previous studies have shown that in addition to estrogen levels, CVD may also be influenced by other reproductive factors, including parity, lipid levels after childbirth, and miscarriage or recurrent miscarriage [8–11]. Bridger Staatz C et al. also found that a variety of family factors, including the number of children, the age at first birth (AFB) and age of last birth (ALB), and the birth interval have been connected obesity and overweight [12]. And, obesity and overweight are commonly associated with an increased risk of chronic diseases, especially CVD [13]. Meanwhile, pregnancy plays a crucial role in determining a woman’s long-term health. During pregnancy, a mother’s body experiences hormonal, immune, and metabolic changes to facilitate healthy fetal growth [14]. Lind JM et al. observed that hypertension treatment rates were lower among women who were older at the time of their first delivery compared to younger mothers [15]. Therefore, we hypothesized that female reproductive factors (AFB, ALB, number of pregnancies and live births) possibly influences the occurrence of clinical cardiovascular events. Currently, understanding of the impact of AFB, ALB, and number of pregnancies, and live births with risk of CVD is still limited. Therefore, we utilized the National Health and Nutrition Examination Survey (NHANES) database 1999–2018 years to investigate the association between AFB, ALB, and number of pregnancies, and live births with history of CVD.

## Material and methods

### Study population

**Fig. 1:**
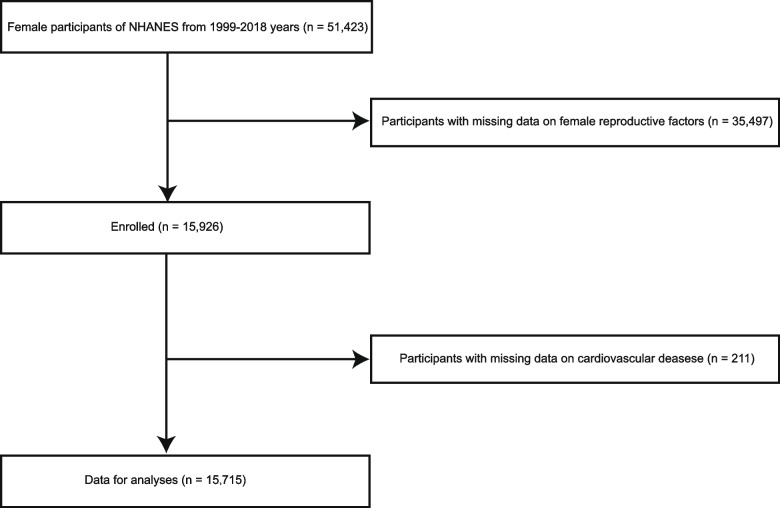
Study flow chart. Abbreviations: NHANES, National Health and Nutrition Examination Surveys; AFB, age at first birth; CVD, cardiovascular disease

The NHANES database is a complex survey that combines interviews and physical examinations to obtain a nationally representative sample of the civilian, noninstitutionalized United States (U.S.) population [16]. In this study, we analysed NHANES data from ten two-year cycles (1999–2018). Among the 51,423 women participants in the total sample, there were 35,497 without AFB, ALB, number of pregnancies, or live births. In addition, we also excluded female participants with missing CVD data (*n* = 211). Finally, a total of 15,715 women who were aged 20 years or over were included in this study (Fig. 1). All the NHANES procedures were approved by the National Center for Health Statistics Institutional Review Board, and all participants involved have signed informed consent [17]. The NHANES website (https://www.cdc.gov/nchs/nhanes/) contains complete information about the survey design, methodology, and data.

### Reproductive factors

Self-administered questionnaires were used to assess reproductive factors. Through the reproductive health questionnaires, women participants recalled their AFB, ALB, time of pregnancy, time of live birth, age at menopause, menopause status, whether to use oral contraceptives, whether to use female hormones, whether to have a hysterectomy, whether to remove both ovaries, and fertile lifespan at the time of the survey. The fertile lifespan was calculated by subtracting the age at menopause from the age at menarche. Those who had previous pregnancy losses (prior stillbirth, miscarriage, or ectopic pregnancy) were not included in this research since AFB only pertains to the first live birth. Female participants had used estrogen or progesterone, including any forms of female hormones, such as pills, creams, patches, and injectables, but did not include birth control methods or use for infertility. More details are available on the NHANES website (https://wwwn.cdc.gov/nchs/nhanes/Default.aspx).

### CVD ascertainment

The primary outcome for the study was CVD which defined as a composite of five self-reported outcomes (congestive heart failure (CHF), coronary heart disease (CHD), angina pectoris, heart attack and stroke) [18]. The participant will be considered patients with CVD if she/he replied “yes” to the question: “Has a doctor or other health professional ever told you that you had CHF/heart attack/CHD/angina pectoris/stroke?”. A standardized medical condition questionnaire administered during the personal interview provides more detailed information (www.cdc.gov/nchs/nhanes/).

### Covariates

The NHANES database provides information and downloads of the following covariates: age, sex, the complication of diabetes mellitus (DM), race/ethnicity, education level, the complication of hypertension, total cholesterol (TC), family poverty income ratio (PIR), body mass index (BMI), waist circumference, marital status, blood urea nitrogen (BUN), smoker, work activity, mean energy intake, hemoglobin (Hb), serum creatinine (Scr), high-density lipoprotein-cholesterol (HDL-C), recreational activity, triglyceride (TG), serum uric acid (sUA), drinker, and estimated glomerular filtration rate (eGFR). For the study, family PIR was used to create two categories of income status: low (family PIR < 1.3) and mid-high (family PIR ≥ 1.3) [19]. Individuals who had smoked less than 100 cigarettes in their lifetime, do not smoke at present, smoked more than 100 cigarettes in their lifetime and smoked some days or every day were defined as non-smokers, former smokers, and current smokers, respectively. There were five categories of drinkers: non-drinkers were defined as had < 12 drinks in lifetime; former drinkers were defined as had ≥ 12 drinks in 1 year and did not drink last year, or did not drink last year but drank ≥ 12 drinks in lifetime; current heavy alcohol consumption was defined as ≥ 3 drinks per day for females, or binge drinking (≥ 4 drinks on the same occasion for females) on five or more days per month; current moderate alcohol consumption was defined as ≥ 2 drinks per day for females, or binge drinking ≥ 2 days per month; and current mild alcohol use was defined as not meeting the above criteria [20]. After resting quietly in a sitting position for 5 min and determining the maximum inflation level, three consecutive blood pressure readings are obtained. If a blood pressure measurement is interrupted or incomplete, a fourth attempt may be made. Hypertension was defined as three consecutive average systolic blood pressure greater than 140 mmHg, or diastolic blood pressure greater than 90 mmHg, or self-reported hypertension or self-reported use of antihypertensive medication [21]. DM was defined as self-reported doctor diagnosis of diabetes, use of insulin or oral hypoglycaemic medication, fasting blood glucose ≥ 7.0 mmol/L (126 mg/dL), postprandial 2-h plasma glucose ≥ 11.1 mmol/L (200 mg/dL) from an oral glucose tolerance test, or glycated haemoglobin A1c (HbA1c) ≥ 6.5% (48 mmol/mol) [22]. Physical activity levels, including recreational activity and work activity, were assessed using questions from the separate questionnaires, including time spent sitting and time spent engaged in typical physical activity over the past week [23]. If the participant satisfied the vigorous physical activity recommendation (minimum 20 min of vigorous physical activity a day, at least three times a week), then it was coded as “Yes”, or if he did not as “No”, respectively, for work and recreational activity [24]. Details of all variables are available online at https://www.cdc.gov/nchs/nhanes/.

### Sample weight computation

All NHANES estimations were based on sample weights computed [25]. The ‘survey’ package was used for sample weight computation [26]. The questionnaire was administered first, and the total population of the United States/the number of people who participated in the questionnaire = wtint2yr. Among them, the years 1999–2000 and 2001–2002 had one wtint2yr each, but were combined to have one wtint4yr. In this study, we selected 10 years of survey samples for merging and then conducted data analysis survey. The survey data of every 2 years have corresponding weights. After reasonable selection of weights, the combined years can be according to the following formula: $Weight=\frac{2}{10}$*(wtint4yr (1999–2000) + wtint4yr (2001–2002) + $\frac{1}{10}$*(wtint2yr (2003–2004) + wtint2yr (2005–2006) + wtint2yr (2007–2008) + wtint2yr (2009–2010) + wtint2yr (2011–2012) + wtint2yr (2013–2014) + wtint2yr (2015–2016) + wtint2yr (2017–2018)).

### Statistical analysis

The required covariates presented in the methodology section had missing values, but the proportion of missing values was less than 10%. We used the ‘mice’ package in the R language for multiple imputation. Multiple imputation method selection: predicted mean matched multiple imputation method, the number of imputations was 20 [27]. The means (standard deviations, SDs) were presented for continuous variables, while numbers and percentages (%) were used to express categorical variables [28]. To calculate differences between groups, we used weighted t-tests (continuous variables) and weighted chi-square tests (categorical variables). Weighted multivariable logistic regression models were used to investigate the association between AFB, ALB, and history of CVD [29]. We used the ‘svyglm’ function in the “survey” package to construct three models: model 1, which adjusted for age and sex; model 2, which adjusted for age, educational level, drinking, marital status, race/ethnicity, sex, smoking, family PIR, the complication of hypertension, and DM; and model 3, which adjusted for all of the potential confounding factors listed in Table 1. All the analyses were performed with R version 3.6.4 and SPSS version 22.0 software. *P-*value < 0.05 was regarded as statistically significant. Restricted cubic plots (RCS) were used to fit nonlinear relationships between independent and dependent variables. The spline curve is essentially a continuous smooth piecewise cubic polynomial, which is limited by some control points, called “nodes”. The “nodes” are placed at multiple locations within the data range, and the type of polynomial and the number and location of nodes determine the type of spline. The “cubed” means that the function is a polynomial of degree 3. The “limitation” is the additional requirement on the basis of the regression splines: the spline function is linear within two intervals [X1, X2) and (xn-1, Xn] at both ends of the data range of the independent variables [30]. The number of RCS nodes is more important than the location. Since the selection of the number of nodes is related to the degree of freedom, more nodes can be taken when the sample size is relatively large [31]. However, the more nodes, the more degrees of freedom, and the more complex the model. It is generally recommended that when the number of nodes is 4, the model fitting effect is better, that is, at the same time, the smoothness of the curve can be taken into account and the accuracy reduction caused by over-fitting can be avoided [32]. Therefore, we take four nodes to fit the association between female reproductive factors (AFB, ALB, number of pregnancies, and live births) and history of cardiovascular disease (CVD) in this study. Sensitivity analysis is a method that evaluates the robustness of a method by changing the method, model, unmeasured variable values, and assumptions. In essence, the assumption conditions and statistical methods are changed and the statistical analysis is performed again to determine whether the results have changed. The purpose is to examine the stability of the results and enhance the credibility of the conclusions. In order to assess if the results we obtained using this full sample set were biased by the presence of missing values. Sensitivity analyses were also performed with the use of data with missing covariates.

**Table 1:** Demographic characteristics of the study women in the United States from NHANES 1999–2018

Variables	Overall (*n* = 15,715)	Non-CVD (*n* = 13,997)	CVD (*n* = 1,718)	*P*-value (Adjusted)
Age, years	52.29 ± 0.19	50.88 ± 0.19	65.62 ± 0.45	< 0.001 (< 0.001)
Race, *n* (%)				< 0.001 (< 0.001)
Mexican American	3068 (19.5%)	2872 (18.3%)	196 (1.2%)	
Other Hispanic	1406 (8.9%)	1283 (8.2%)	123 (0.8%)	
Non-Hispanic Black	3217 (20.5%)	2815 (17.9%)	402 (2.6%)	
Non-Hispanic White	6842 (43.5%)	5934 (37.8%)	908 (5.8%)	
Other race	1182 (7.5%)	1093 (7.0%)	89 (0.6%)	
Family PIR				< 0.001 (< 0.001)
< 1.3	5299 (35.0%)	4772 (30.4%)	727 (4.6%)	
≥ 1.3	10,216 (65.0%)	9225 (58.7%)	991 (6.3%)	
Education level, *n* (%)				< 0.001 (< 0.001)
Less than high school	4700 (29.9%)	4071 (25.9%)	629 (4.0%)	
High school	1528 (9.7%)	1316 (8.4%)	212 (1.3%)	
More than high school	9487 (60.4%)	8610 (54.8%)	877 (5.6%)	
Marital status, *n* (%)				< 0.001 (< 0.001)
Having a partner	9358 (59.5%)	8645 (55.0%)	713 (4.5%)	
No partner	5219 (33.2%)	4304 (27.4%)	915 (5.8%)	
Unmarried	1138 (7.2%)	1048 (6.7%)	90 (0.6%)	
Hypertension, *n* (%)				< 0.001 (< 0.001)
No	8086 (51.5%)	7820 (49.8%)	266 (1.7%)	
Yes	7629 (48.5%)	6177 (39.3%)	1452 (9.2%)	
DM, n (%)				< 0.001 (< 0.001)
No	12,741 (81.1%)	11,698 (74.4%)	1043 (6.6%)	
Yes	2974 (18.9%)	2299 (14.6%)	675 (4.3%)	
Smoker, *n* (%)				< 0.001 (< 0.001)
No	9824 (62.5%)	8943 (56.9%)	881 (5.6%)	
Former	3152 (20.1%)	2655 (16.9%)	497 (3.2%)	
Now	2739 (17.4%)	2399 (15.3%)	340 (2.2%)	
Alcohol user, *n* (%)				< 0.001 (< 0.001)
No	3548 (22.6%)	3103 (19.8%)	445 (2.8%)	
Former	2954 (18.8%)	2431 (15.5%)	523 (3.3%)	
Mild	4460 (28.4%)	4025 (25.6%)	435 (2.8%)	
Moderate	2659 (16.9%)	2482 (15.8%)	177 (1.1%)	
Heavy	2094 (13.3%)	1956 (12.4%)	138 (0.9%)	
Menopause status, *n* (%)				< 0.001 (< 0.001)
No	6779 (43.1%)	6488 (41.3%)	291 (1.9%)	
Yes	8936 (56.9%)	7509 (47.8%)	1427 (9.1%)	
Oral contraceptive use, *n*(%)				< 0.001 (< 0.001)
No	5390 (34.3%)	4581 (29.2%)	809 (5.1%)	
Yes	10,325 (65.7%)	9416 (59.9%)	909 (5.8%)	
Use female hormones, *n* (%)				< 0.001 (< 0.001)
No	11,987 (76.3%)	10,843 (69.0%)	1144 (7.3%)	
Yes	3728 (23.7%)	3154 (20.0%)	574 (3.7%)	
Had a hysterectomy, *n* (%)				< 0.001 (< 0.001)
No	11,304 (71.9%)	10,424 (66.3%)	880 (5.6%)	
Yes	4411 (28.1%)	3573 (22.7%)	838 (5.3%)	
Both ovaries removed, *n* (%)				< 0.001 (< 0.001)
No	13,056 (83.1%)	11,859 (75.5%)	1197 (7.6%)	
Yes	2659 (16.9%)	2138 (13.6%)	521 (3.3%)	
Work activity, *n* (%)				< 0.001 (< 0.001)
No	9225 (58.7%)	8070 (51.4%)	1155 (7.3%)	
Yes	6490 (41.3%)	5927 (37.7%)	563 (3.6%)	
Recreational activity, *n* (%)				< 0.001 (< 0.001)
No	10,556 (67.2%)	9208(58.6%)	1348 (8.6%)	
Yes	5159 (32.8%)	4789 (34.2%)	370 (2.4%)	
CHD, *n* (%)				-
No	15,214 (96.8%)	13,997 (89.1%)	1217 (7.7%)	
Yes	501 (3.2%)	0 (0.0%)	501 (3.2%)	
CHF, *n* (%)				-
No	15,215 (96.8%)	13,997 (89.1%)	1218 (7.8%)	
Yes	500 (3.2%)	0 (0.0%)	500 (3.2%)	
Angina pectoris, *n* (%)				-
No	15,253 (97.1%)	13,997 (89.1%)	1256 (8.0%)	
Yes	462 (2.9%)	0 (0.0%)	462 (2.9%)	
Heart attack, *n* (%)				-
No	15,166 (96.5%)	13,997 (89.1%)	1169 (7.4%)	
Yes	549 (3.5%)	0 (0.0%)	549 (3.5%)	
Stroke, *n* (%)				-
No	15,041 (95.7%)	13,997 (89.1%)	1044 (6.6%)	
Yes	674 (4.3%)	0 (0.0%)	674 (4.3%)	
BMI, kg/m^2^	29.34 ± 0.09	29.18 ± 0.10	30.80 ± 0.24	< 0.001 (< 0.001)
Waist circumference, cm	97.38 ± 0.22	96.83 ± 0.23	102.62 ± 0.55	< 0.001 (< 0.001)
Hb, g/dL	13.47 ± 0.02	13.48 ± 0.02	13.36 ± 0.05	0.004 (0.008)
Mean energy intake (kcal/day)	1762.92 ± 6.96	1784.32 ± 7.34	1559.93 ± 18.01	< 0.001 (< 0.001)
TC, mg/dL	202.10 ± 0.52	202.68 ± 0.54	196.68 ± 1.35	< 0.001 (< 0.001)
TG, mg/dL	126.94 ± 1.03	124.91 ± 1.08	146.22 ± 2.71	< 0.001 (< 0.001)
HDL-C, mg/dL	58.11 ± 0.24	58.40 ± 0.25	55.39 ± 0.49	< 0.001 (< 0.001)
BUN, mg/dL	13.34 ± 0.08	12.97 ± 0.08	16.83 ± 0.23	< 0.001 (< 0.001)
UA, mg/dL	4.84 ± 0.01	4.77 ± 0.01	5.52 ± 0.05	< 0.001 (< 0.001)
Scr, mg/dL	0.78 ± 0.00	0.76 ± 0.00	0.98 ± 0.02	< 0.001 (< 0.001)
eGFR, ml/min/1.73m^2^	91.02 ± 0.32	93.04 ± 0.32	71.90 ± 0.81	< 0.001 (< 0.001)
AFB, years	22.61 ± 0.09	22.75 ± 0.10	21.26 ± 0.13	< 0.001 (< 0.001)
ALB, years	29.18 ± 0.08	29.16 ± 0.08	29.39 ± 0.21	0.267 (0.534)
Number of live births, times	2.86 ± 0.02	2.80 ± 0.02	3.40 ± 0.06	< 0.001 (< 0.001)
Number of pregnancies, times	3.60 ± 0.02	3.54 ± 0.02	4.18 ± 0.06	< 0.001 (< 0.001)
Age at menarche, years	12.74 ± 0.02	12.74 ± 0.02	12.76 ± 0.05	0.796 (0.999)
Age at menopause, years	41.74 ± 0.13	41.59 ± 0.13	43.12 ± 0.28	< 0.001 (< 0.001)
Fertile lifespan, years	29.00 ± 0.13	28.85 ± 0.14	30.37 ± 0.29	< 0.001 (< 0.001)

Data are presented as mean ± *SD* or *n* (%)

﻿*CVD* Cardiovascular disease, *DM* Diabetes mellitus, *BMI* Body mass index, *Hb* Hemoglobin, *CHD* Coronary heart disease, *CHF* Congestive heart failure, *BUN* Blood urea nitrogen, *UA* Uric acid, *Scr* Serum creatinine, *TC* Total cholesterol, *TG* Triglycerides, *HDL-cholesterol* High density lipoprotein-cholesterol, *eGFR* Estimated glomerular filtration rate, *AFB* Age at first birth, *ALB* Age at last birth

## Results

### Baseline characteristics

The baseline characteristics of the participants involved in the current study are depicted in Table 1. In total, 15,715 people (aged 52.29 ± 0.19 years) were included in our study. CVD was present in 10.9% of this population. Significant differences were found in baseline characteristics between the CVD group and the non-CVD group, with the exception of the ALB and age at menarche (*P*-value < 0.05). Finally, we also compared the characteristics of the populations between the those with missing values and those without in Supplementary Table 1.

### Association between AFB, ALB, number of pregnancies and live births and total CVD

**Fig. 2:**
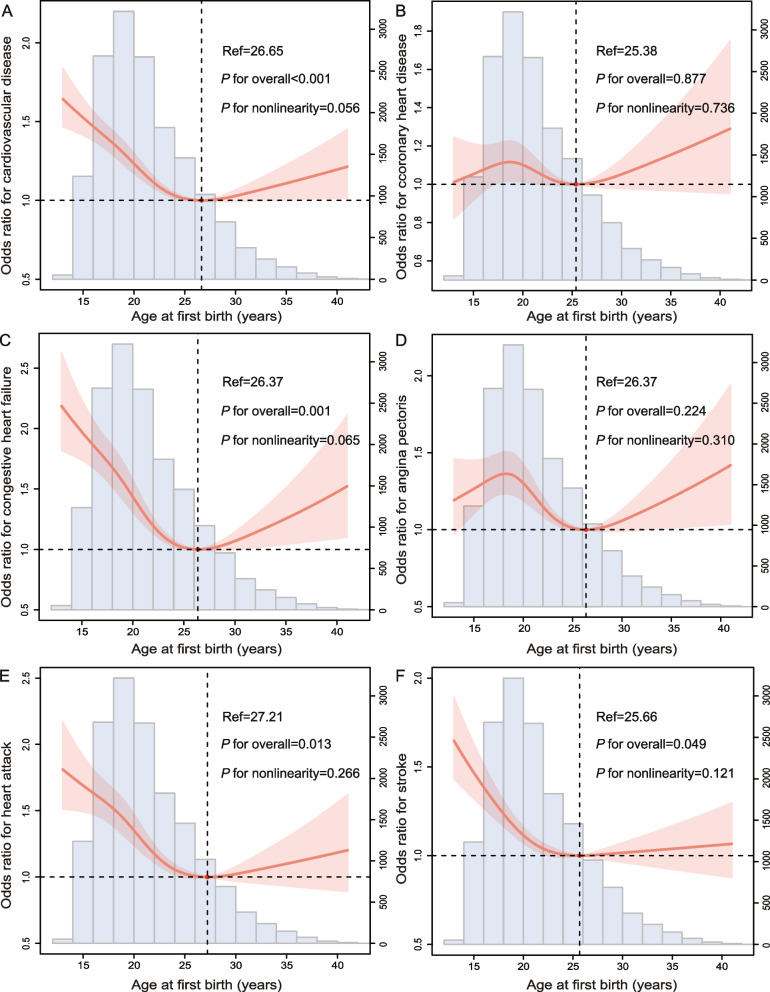
The RCS curve of the association between AFB and **A** total CVD, **B** CHD, **C** CHF, **D** angina pectoris, **E** heart attack, and **F** stroke in women in the United States from NHANES 1999–2018. Abbreviation: RCS, restricted cubic spline; AFB, age at first birth; CVD, cardiovascular disease; CHD, coronary heart disease; CHF, congestive heart failure

**Fig. 3:**
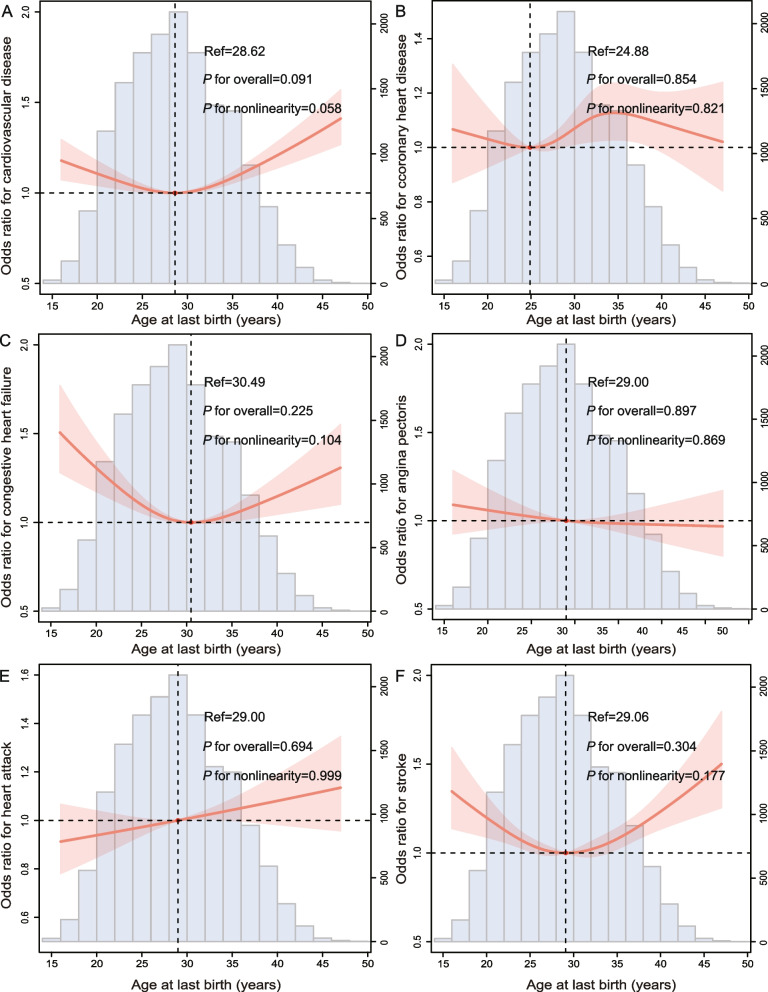
The RCS curve of the association between ALB and **A** total CVD, **B** CHD, **C** CHF, **D** angina pectoris, **E** heart attack, and **F** stroke in women in the United States from NHANES 1999–2018. Abbreviation: RCS, restricted cubic spline; ALB, age at last birth; CVD, cardiovascular disease; CHD, coronary heart disease; CHF, congestive heart failure

**Fig. 4:**
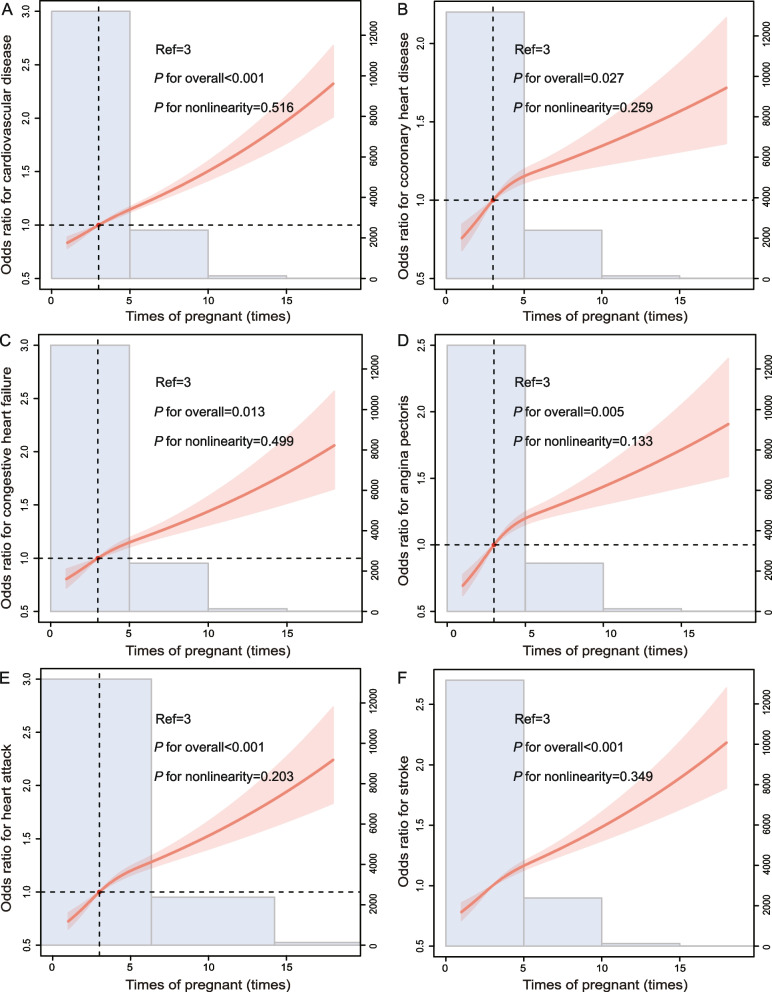
The RCS curve of the association between number of pregnancies and **A** total CVD, **B** CHD, **C** CHF, **D** angina pectoris, **E** heart attack, and **F** stroke in women in the United States from NHANES 1999–2018. Abbreviation: RCS, restricted cubic spline; CVD, cardiovascular disease; CHD, coronary heart disease; CHF, congestive heart failure

**Fig. 5:**
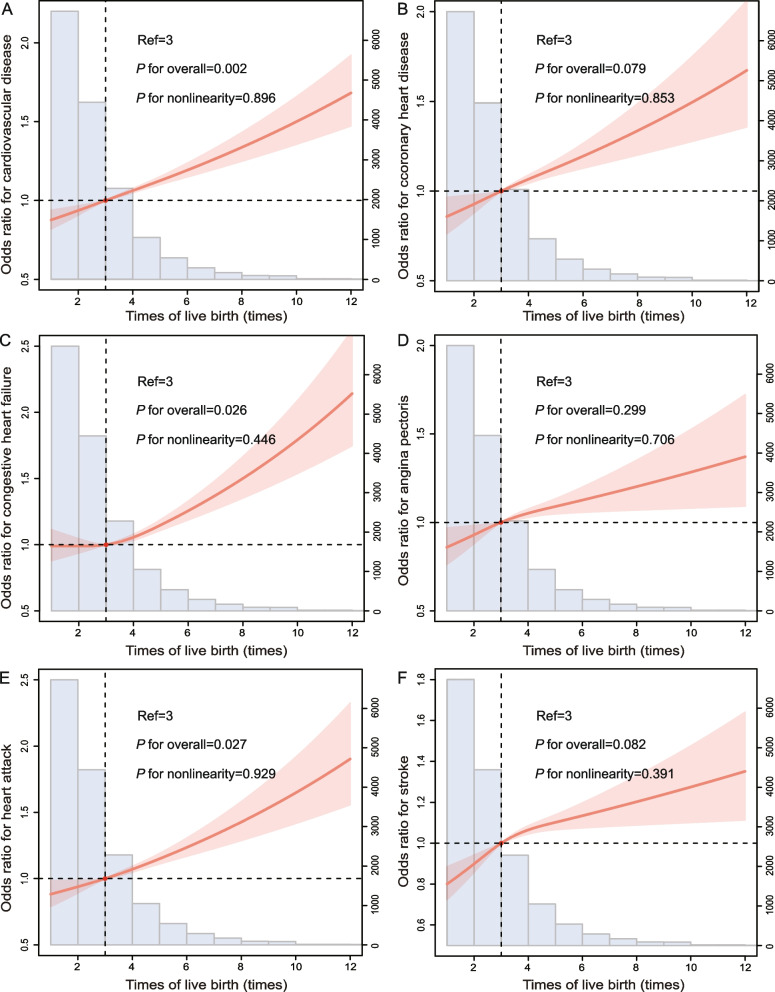
The RCS curve of the association between number of live births and **A** total CVD, **B** CHD, **C** CHF, **D** angina pectoris, **E** heart attack, and **F** stroke in women in the United States from NHANES 1999–2018. Abbreviation: RCS, restricted cubic spline; CVD, cardiovascular disease; CHD, coronary heart disease; CHF, congestive heart failure

The AFB, and ALB displayed a U-shaped relationship with the history of total CVD (Figs. 2A, and 3A). However, there was a positive linear correlation between the number of pregnancies and live births and history of total CVD (Figs. 4A, and 5A). Women participants were divided into four groups based on AFB (< 25; 25–27; 28–34; and > 34 years), with 25–27 years as the reference group. And, they were also grouped into four groups based on ALB (< 25; 25–29; 30–34; and > 34 years), with 25–29 years as the reference group. Table 2 displayed the results of weighted multivariate logistic regression analyses for the association of AFB and ALB with history of total CVD. After controlling for underlying cofounders, compared to women with AFB of 25–27 years old (reference group), the odds ratios (ORs) with 95% confidence intervals (CIs) of history of total CVD were 1.24 (1.03, 1.49), 1.10 (0.92, 1.45), and 1.15 (0.94, 1.37) for < 25, 28–34, and > 34 years old group. In addition, in comparison to the ALB of 25–29 years old, the odds ratios with 95% CIs for history of total CVD were 1.13 (0.96, 1.33), 1.06 (0.91, 1.23), and 1.17 (0.99, 1.37) for < 25, 30–34, and > 34 years old group, respectively, in the fully-adjusted model. Additionally, women participants also were divided into four groups based on number of pregnancies, and live births (0–2; 3; 4; and > 5 times), with 0–2 as the reference group. Table 3 also displayed the results of weighted multivariate logistic regression analyses for the association of number of pregnancies, and live births with history of total CVD.

**Table 2:** Associations of AFB, and ALB with history of total CVD in women in the United States from NHANES 1999–2018

	Model 1		Model 2		Model 3	
	OR (95%CI)	*P* for trend (Adjusted)	OR (95%CI)	*P* for trend (Adjusted)	OR (95%CI)	*P* for trend (Adjusted)
AFB		< 0.001 (< 0.001)		< 0.001 (< 0.001)		0.049 (0.098)
25–27 (2046)	1.00		1.00		1.00	
< 25 (11,673)	1.65 (1.38, 1.96) ^***^		1.35 (1.13, 1.62) ^**^		1.24 (1.03, 1.49) ^*^	
28–34 (1736)	1.09 (0.87, 1.37)		1.01 (0.86, 1.33)		1.10 (0.92, 1.45)	
> 34 (260)	1.32 (1.07, 1.58) ^*^		1.19 (0.91, 1.34)		1.15 (0.94, 1.37)	
ALB		< 0.001 (< 0.001)		< 0.001 (< 0.001)		0.020 (0.040)
25–29 (4708)	1.00		1.00		1.00	
< 25 (3479)	1.33 (1.14, 1.55) ^***^		1.16 (0.99, 1.36)		1.13 (0.96, 1.33)	
30–34 (4236)	1.03 (0.90, 1.17)		1.01 (0.87, 1.18)		1.06 (0.91, 1.23)	
> 34 (3292)	1.10 (0.97, 1.26)		1.08 (0.94, 1.23)		1.17 (0.99, 1.37)	

Model 1: age and race/ethnicity. Model 2: model 1 variables plus education level, marital status, family poverty-income ratio, hypertension, diabetes mellitus, smoker, alcohol user; Model 3 was adjusted for model 2 variables plus body mass index, waist circumference, mean energy intake, hemoglobin, fast glucose, glycosylated hemoglobin, menopause status, oral contraceptive use, use female hormones, had a hysterectomy, both ovaries removed, blood urea nitrogen, uric acid, serum creatinine, estimated glomerular filtration rate, total cholesterol, triglyceride, high-density lipoprotein-cholesterol, time of live birth, time of pregnant, age at menarche, age at menopause, and fertile lifespan. Of these, 13,997 were non-CVD and 1,718 were CVD

*AFB* Age at first birth, *ALB* Age at last birth, *CVD* Cardiovascular disease, *OR* Odd ratio, *CI* Confidence interval

^*^P < 0.05, ^**^P < 0.01, ^***^P < 0.001

**Table 3:** Associations of number of pregnancies, and live births with history of total CVD in women in the United States from NHANES 1999–2018

	Model 1		Model 2		Model 3	
	OR (95%CI)	*P* for trend (Adjusted)	OR (95%CI)	*P* for trend (Adjusted)	OR (95%CI)	*P* for trend (Adjusted)
Number of pregnancies		< 0.001 (< 0.001)		0.003 (0.006)		0.004 (0.008)
0–2 (4225)	1.00		1.00		1.00	
3 (4122)	1.02 (0.84, 1.15)		1.03 (0.83, 1.14)		1.05 (0.80, 1.11)	
4 (3141)	1.16 (0.99, 1.36)		1.10 (0.93, 1.30)		1.07 (0.90, 1.26)	
> 5 (4227)	1.39 (1.21, 1.61) ^***^		1.21 (1.04, 1.40) *		1.20 (1.04, 1.40)	
Number of Live births		< 0.001 (< 0.001)		0.009 (0.018)		0.012 (0.024)
0–2 (6849)	1.00		1.00		1.00	
3 (4444)	1.11 (0.97, 1.27)		1.08 (0.94, 1.24)		1.05 (0.91, 1.21)	
4 (2282)	1.32 (1.13, 1.54) ^***^		1.17 (0.99, 1.37)		1.14 (0.97, 1.34)	
> 5 (2140)	1.48 (1.27, 1.72) ^***^		1.22 (1.04, 1.43) ^*^		1.22 (1.03, 1.43) ^*^	

Model 1: age and race/ethnicity. Model 2: model 1 variables plus education level, marital status, family poverty-income ratio, hypertension, diabetes mellitus, smoker, alcohol user; Model 3 was adjusted for model 2 variables plus body mass index, waist circumference, mean energy intake, hemoglobin, fast glucose, glycosylated hemoglobin, menopause status, oral contraceptive use, use female hormones, had a hysterectomy, both ovaries removed, blood urea nitrogen, uric acid, serum creatinine, estimated glomerular filtration rate, total cholesterol, triglyceride, high-density lipoprotein-cholesterol, time of live birth, time of pregnant, age at menarche, age at menopause, and fertile lifespan. Of these, 13,997 were non-CVD and 1,718 were CVD

*CVD* Cardiovascular disease, *OR* Odd ratio, *CI* Confidence interval

^*^P < 0.05, ^***^P < 0.001

### Association between AFB, ALB, and number of pregnancies and live births and individual CVDs

Individual CVDs include angina pectoris, CHD, heart attack, CHF, and stroke. And, we analysed the relationship between them and AFB or ALB separately (Figs. 2, 3, 4, and 5). A U-curve association between AFB and history of individuals’ CVD, including CHF, heart attack, and stroke, was exhibited in the RCS plot (Fig. 2C, E, and F). In addition, with the increase in AFB, the history of CHD and angina pectoris showed a trend of first increasing, then decreasing, and then increasing (Fig. 2B and D). Meanwhile, there was a U-shaped link between ALB and history of individual CVDs, including CHF and stroke (Fig. 3C and F). However, there was first a decrease in correlations between ALB and CHD, then an increase, and finally another decrease (Fig. 3B). In addition, the ALB was linearly negative with angina pectoris (Fig. 3D) but linearly positive with a heart attack (Fig. 3E). In addition, the number of pregnancies and live births was positively correlated with history of individuals’ CVD, including CHD, CHF, angina, heart attack, and stroke (Figs. 4B-F and 5B-F). The results of weighted multivariable logistic regression models further showed the association between AFB and ALB and history of individual CVDs, including CHD, CHF, angina pectoris, heart attack, and stroke (Tables 4, 5, 6 and 7).

**Table 4:** Associations of AFB with history of individual CVD in women in the United States from NHANES 1999–2018

AFB	CHD	CHF	Angina pectoris	Heart attack	Stroke
	OR (95%CI)	OR (95%CI)	OR (95%CI)	OR (95%CI)	OR (95%CI)
25–27 (2046)	1.00	1.00	1.00	1.00	1.00
< 25 (11,673)	1.09 (0.81, 1.48)	1.43 (0.90, 1.92)	1.37 (0.97, 1.92)	1.48 (1.07, 2.05) ^*^	1.16 (0.86, 1.49)
28–34 (1736)	1.05 (0.76, 1.55)	1.17 (0.74, 1.66)	1.14 (0.91, 1.39)	1.15 (0.79, 1.76)	1.01 (0.78, 1.31)
> 34 (260)	1.22 (0.89, 1.53)	1.34 (0.96, 1.85)	1.40 (0.94, 1.94)	1.24 (0.47, 3.23)	1.06 (0.88, 1.27)
*P* for trend (Adjusted)	0.689 (0.999)	0.920 (0.999)	0.251 (0.502)	0.294 (0.588)	0.353 (0.706)

Analysis was adjusted for age, race/ethnicity, education level, marital status, family poverty-income ratio, hypertension, diabetes mellitus, smoker, alcohol user, body mass index, waist circumference, mean energy intake, hemoglobin, fast glucose, glycosylated hemoglobin, menopause status, oral contraceptive use, use female hormones, had a hysterectomy, both ovaries removed, blood urea nitrogen, uric acid, serum creatinine, estimated glomerular filtration rate, total cholesterol, triglyceride, high-density lipoprotein-cholesterol, time of live birth, time of pregnant, age at menarche, age at menopause, and fertile lifespan. Of these, 15,214 women were non-CHD and 501 women were CHD; 15,215 women were non-CHF and 500 women were CHF; 15,253 women were angina pectoris and 462 women were non-angina pectoris; 15,166 were non-heart attack and 549 women were heart attack; 15,041 were non-stroke and 674 women were stroke

*CVD* Cardiovascular disease, *AFB* Age at first birth, *CHD* Coronary heart disease, *CHF* Congestive heart failure, *OR* Odd ratio, *CI* Confidence interval

^*^P < 0.05

**Table 5:** Associations of ALB with the history of individual CVD in women in the United States from NHANES 1999–2018

ALB	CHD	CHF	Angina pectoris	Heart attack	Stroke
	OR (95%CI)	OR (95%CI)	OR (95%CI)	OR (95%CI)	OR (95%CI)
25–29 (4708)	1.00	1.00	1.00	1.00	1.00
< 25 (3479)	1.09 (0.82, 1.45)	1.15 (0.88, 1.50)	1.27 (0.96, 1.67)	0.91 (0.66, 1.34)	1.14 (0.91, 1.44)
30–34 (4236)	1.13 (0.87, 1.45)	0.89 (0.68, 1.15)	0.94 (0.76, 1.21)	1.05 (0.83, 1.35)	1.07 (0.85, 1.33)
> 34 (3292)	1.11 (0.85, 1.45)	1.27 (0.75, 1.66)	0.87 (0.61, 1.14)	1.15 (0.89, 1.48)	1.14 (0.90, 1.43)
*P* for trend (Adjusted)	0.387 (0.774)	0.496 (0.992)	0.438 (0.876)	0.347 (0.694)	0.351 (0.702)

Analysis was adjusted for age, race/ethnicity, education level, marital status, family poverty-income ratio, hypertension, diabetes mellitus, smoker, alcohol user, body mass index, waist circumference, mean energy intake, hemoglobin, fast glucose, glycosylated hemoglobin, menopause status, oral contraceptive use, use female hormones, had a hysterectomy, both ovaries removed, blood urea nitrogen, uric acid, serum creatinine, estimated glomerular filtration rate, total cholesterol, triglyceride, high-density lipoprotein-cholesterol, time of live birth, time of pregnant, age at menarche, age at menopause, and fertile lifespan. Of these, 15,214 women were non-CHD and 501 women were CHD; 15,215 women were non-CHF and 500 women were CHF; 15,253 women were angina pectoris and 462 women were non-angina pectoris; 15,166 were non-heart attack and 549 women were heart attack; 15,041 were non-stroke and 674 women were stroke

*CVD* Cardiovascular disease, *ALB* Age at last birth, *CHD* Coronary heart disease, *CHF* Congestive heart failure, *OR* Odd ratio, *CI* Confidence interval

**Table 6:** Associations of number of pregnancies with history of individual CVD in women in the United States from NHANES 1999–2018

Number of pregnancies	CHD	CHF	Angina pectoris	Heart attack	Stroke
	OR (95%CI)	OR (95%CI)	OR (95%CI)	OR (95%CI)	OR (95%CI)
0–2 (4225)	1.00	1.00	1.00	1.00	1.00
3 (4122)	1.05 (0.81, 1.26)	1.06 (0.82, 1.27)	1.01 (0.75, 1.35)	1.01 (0.78, 1.32)	1.04 (0.82, 1.33)
4 (3141)	1.18 (0.88, 1.56)	1.11 (0.83, 1.47)	1.09 (0.80, 1.47)	1.17 (0.89, 1.53)	1.09 (0.85, 1.40)
> 5 (4227)	1.28 (0.99, 1.66)	1.22 (0.95, 1.57)	1.54 (1.18, 2.01) ^**^	1.21 (0.95, 1.55)	1.35 (1.08, 1.69) ^*^
*P*for trend (Adjusted)	0.020 (0.040)	0.065 (0.130)	< 0.001 (< 0.001)	0.072 (0.144)	0.005 (0.010)

Analysis was adjusted for age, race/ethnicity, education level, marital status, family poverty-income ratio, hypertension, diabetes mellitus, smoker, alcohol user, body mass index, waist circumference, systolic blood pressure, diastolic blood pressure, mean energy intake, hemoglobin, fast glucose, glycosylated hemoglobin, menopause status, oral contraceptive use, use female hormones, had a hysterectomy, both ovaries removed, blood urea nitrogen, uric acid, serum creatinine, estimated glomerular filtration rate, total cholesterol, triglyceride, high-density lipoprotein-cholesterol, time of live birth, time of pregnant, age at menarche, age at menopause, and fertile lifespan. Of these, 15,214 women were non-CHD and 501 women were CHD; 15,215 women were non-CHF and 500 women were CHF; 15,253 women were angina pectoris and 462 women were non-angina pectoris; 15,166 were non-heart attack and 549 women were heart attack; 15,041 were non-stroke and 674 women were stroke

﻿*CVD* Cardiovascular disease, *AFB* Age at first birth, *CHD* Coronary heart disease, *CHF* Congestive heart failure, *OR* Odd ratio, *CI* Confidence interval

^*^P < 0.05; ^**^P < 0.01

**Table 7:** Associations of number of live births with the history of individual CVD in women in the United States from NHANES 1999–2018

Number of live births	CHD	CHF	Angina pectoris	Heart Attack	Stroke
	OR (95%CI)	OR (95%CI)	OR (95%CI)	OR (95%CI)	OR (95%CI)
0–2 (6849)	1.00	1.00	1.00	1.00	1.00
3 (4444)	1.07 (0.76, 1.24)	1.04 (0.79, 1.37)	1.03 (0.80, 1.33)	1.02 (0.81, 1.29)	1.21 (0.98, 1.49)
4 (2282)	1.17 (0.89, 1.54)	1.09 (0.71, 1.32)	1.09 (0.82, 1.45)	1.08 (0.75, 1.39)	1.22 (0.96, 1.55)
> 5 (2140)	1.18 (0.90, 1.54)	1.22 (0.95, 1.58)	1.37 (1.04, 1.80) ^*^	1.36 (1.06, 1.76) ^*^	1.26 (0.99, 1.59)
*P* for trend (Adjusted)	0.141 (0.282)	0.077 (0.154)	0.035 (0.070)	0.037 (0.074)	0.068 (0.136)

Analysis was adjusted for age, race/ethnicity, education level, marital status, family poverty-income ratio, hypertension, diabetes mellitus, smoker, alcohol user, body mass index, waist circumference, mean energy intake, hemoglobin, fast glucose, glycosylated hemoglobin, menopause status, oral contraceptive use, use female hormones, had a hysterectomy, both ovaries removed, blood urea nitrogen, uric acid, serum creatinine, estimated glomerular filtration rate, total cholesterol, triglyceride, high-density lipoprotein-cholesterol, time of live birth, time of pregnant, age at menarche, age at menopause, and fertile lifespan. Of these, 15,214 women were non-CHD and 501 women were CHD; 15,215 women were non-CHF and 500 women were CHF; 15,253 women were angina pectoris and 462 women were non-angina pectoris; 15,166 were non-heart attack and 549 women were heart attack; 15,041 were non-stroke and 674 women were stroke

*CVD* Cardiovascular disease, *ALB* Age at last birth, *CHD* Coronary heart disease, *CHF* Congestive heart failure, *OR* Odd ratio, *CI* Confidence interval

^*^P < 0.05

### Sensitivity analysis

To avoid bias caused by differences in these potential biases due to missing as well as age-related non-correspondence, we carried out a sensitivity analysis focused on the inclusion of individuals who were removed because of missing covariates: using this sensitivity analysis the improvement of history of CVD in AFB, ALB, number of pregnancies, and live births remained statistically significant (Supplementary Table 2, 3, 4, 5, 6, and 7; Supplementary Figure 1, 2, 3 and 4).

## Discussion

An analysis of NHANES data (1999–2018) was performed on a large cross-sectional sample of women > 20 years of age in this study. We observed a U-shaped correlation between the AFB and ALB and history of total CVD. However, the history of total CVD and the number of pregnancies and live births were positively linearly related.

In recent years, the average age of parents at the birth of their first child has risen in the United States [33]. Furthermore, the average maternal age at first birth in many European countries is currently high [34]. And, many women deliver their first child after they turn 35 [35]. Earlier maternal age and higher parity at first birth may have long-term effects on the health of women in old age [36]. The effects of pregnancy affect not only systemic vascular resistance and cardiac output but also changes in lipid profiles that may be atherogenic [37]. In addition, the changes in a woman’s physiology and the increase in metabolic demands are caused by pregnancy [38]. Numerous studies have discovered the impact of pregnancy on long-term cardiac status [39–41]. Women who give birth to numerous children and/or at a young age are at a greater risk of developing chronic diseases and poor physical functioning as they age [42]. Chehade H et al. also found that preterm born subjects are exposed to a significantly increased risk for altered cardio-vascular and renal functions at young adulthood [43]. Diabetes is a risk factor for cardiovascular disease [44]. There is also evidence that pregnancy may affect long-term glucose homeostasis and that multiparity is associated with type II diabetes later in life [45–47]. Vandenheede H. and his colleague revealed that having a first child at an early age and multiparity have both been linked with diabetes-related mortality [46]. According to a Korean prospective cohort study, mothers who gave birth at an early or late age were more likely to die from all-cause and CVD mortality than those who gave birth in their mid-20 s [48]. While not all findings are consistent, Wolfson C et al. found no evidence that advanced maternal-age births increased history of CVD later in life in the Nurses’ Health Study II [49]. And, Feldman B. and his co-workers revealed that, when compared to a general population of women, parturient free of major chronic diseases who give birth at a later age do not have increased cardiometabolic outcomes in midlife [50]. It is shown that parity has been linked to CVD—including CHD, heart failure, and strokes-later in life [51]. Numerous large-scale population studies show that a higher number of pregnancies and longer interpregnancy intervals are associated with a higher risk of hypertension and CVD [11]. A study of Aboriginal communities in Australia shows that more than three pregnancies are predisposing to hypertension and heart disease, as well as the Swedish large-scale population linkage study [51]. According to a study in Magnus MC et al., women with a prolonged pregnancy have an increased risk of CVD [52]. As a result, pregnancy may have long-term effects on the cardiovascular system. Many risk factors have been identified through epidemiological research (age at menarche, parity, age at first full-term pregnancy, age at menopause), most of which are related to estrogen production [53]. Feng X and his team found that pregnant women’s estrogen and progesterone levels increase rapidly during pregnancy [54]. The possible mechanism by which female reproductive factors, including AFB, ALB, number of pregnancies, and live births, affect the occurrence of CVD may be related to estrogen.

In addition, we analysed the link between AFB, ALB, the number of pregnancies and live births, and independent cardiovascular adverse events. A U-shaped curve relationship was also observed between AFB and CHF, heart attack, and stroke. Additionally, the association also exists between ALB, CHF, and stroke. The number of pregnancies and live births were all positively correlated with an individual’s CVD risk, including CHD, CHF, angina, heart attack, and stroke. Single-center Japanese research has suggested that women who deliver are more likely to suffer a stroke [55]. A woman of childbearing age has a low stroke risk overall, but the risk spikes during peripartum and early postpartum [56]. The risk of hypertension and other stroke risk factors is increased in adult survivors of preterm birth [57]. Crump C. et al. found that preterm birth was linked with an increased risk of hemorrhagic and ischemic strokes in adulthood in a large Swedish cohort [57]. However, currently, there are no relevant studies exploring the association between AFB and CHF, or heart attack, in the female population in the United States. In addition, studies on the relationship between ALB and CHF are lacking. Additionally, pregnancy complications, including hypertensive disorders of pregnancy and gestational diabetes, may also have a detrimental effect on the occurrence of adverse cardiovascular events [58]. In terms of short- and long-term maternal CVD, hypertensive disorders of pregnancy are significant risk factors [59]. O’Kelly AC has found that women with a history of hypertensive disorders of pregnancy are more likely to have strokes, myocardial infarctions, and cardiomyopathies during the peripartum period than women without a history of hypertensive disorders of pregnancy [60]. In addition, meta-analyses and subsequent large prospective cohort studies have found that women who have had gestational hypertension or preeclampsia in the past have a two-fold increased risk of CVD [61–63]. Among women who have gestational diabetes mellitus, there is a two-fold risk of coronary artery calcium at midlife, regardless of whether they progress to prediabetes or type 2 diabetes mellitus, as well as a 1.5- to 2-fold risk of cardiovascular events [64]. Progression to type 2 diabetes mellitus appears to be associated with a greater risk of CVD events [65]. Additionally, the degree of glucose impairment during pregnancy appears to be related to the risk of subsequent CVD, similar to the relationship between impaired gestational glucose tolerance and type 2 diabetes mellitus [66].

Firstly, the study provides significant reference value for managing childbearing age in women and reducing the risk of CVD by examining the association between AFB, ALB, number of pregnancies and live births, and history of total CVD. Secondly, we used the data from the NHANES database for the 1999–2018 years for our analysis and further conducted subgroup analyses due to the large sample size. This is the main strength of our study. Our study, however, has some limitations. Firstly, this study spans nearly 20 years, which may cause bias due to the fact that people of different ages live in different environments and have different living habits. Secondly, a total of 15,715 women were included in the analysis; that is only 30% of the total sample, which may increase the risk of inclusion bias. Thirdly, we may not have included all the confounding factors, such as pregnancy complications (pre-eclampsia, gestational hypertension, gestational diabetes, and preterm birth), that may influence the results. Fourthly, although the large sample size of this study may have an impact on the statistical significance in the t-test and association analysis. However, these results are still important, even if they are drawn in the context of a large sample size. Finally, self-reported confounders might be susceptible to self-report bias. The conclusions of this study still need to be confirmed by a larger study in other countries.

## Conclusion

Based on the large-scale U.S. general population, our results demonstrate that the early or later AFB and ALB related to the history of CVD in later life for women. Additionally, we should consider focusing on women with earlier or later AFB and ALB for screening and prevention of CVD. Further research is warranted to focus on the potential mechanisms of the relationship between female reproductive factors and the history of CVD.

### Supplementary Information

Supplementary Material 1: Supplementary Figure 1. The RCS curve of the association of AFB with (A) total CVD, (B) CHD, (C) CHF, (D) angina pectoris, (E) heart attack, and (F) stroke. Abbreviation: RCS, restricted cubic spline; AFB, Age at first birth; CVD, cardiovascular disease; CHD, coronary heart disease; CHF, congestive heart failure.

Supplementary Material 2: Supplementary Figure 2. The RCS curve of the association of ALB with (A) total CVD, (B) CHD, (C) CHF, (D) angina pectoris, (E) heart attack, and (F) stroke. Abbreviation: RCS, restricted cubic spline; ALB, Age at last birth; CVD, cardiovascular disease; CHD, coronary heart disease; CHF, congestive heart failure.

Supplementary Material 3: Supplementary Figure 3. The RCS curve of the association of number of pregnancies with (A) total CVD, (B) CHD, (C) CHF, (D) angina pectoris, (E) heart attack, and (F) stroke. Abbreviation: RCS, restricted cubic spline; CVD, cardiovascular disease; CHD, coronary heart disease; CHF, congestive heart failure.

Supplementary Material 4: Supplementary Figure 4. The RCS curve of the association of number of live births with (A) total CVD, (B) CHD, (C) CHF, (D) angina pectoris, (E) heart attack, and (F) stroke. Abbreviation: RCS, restricted cubic spline; CVD, cardiovascular disease; CHD, coronary heart disease; CHF, congestive heart failure.

Supplementary Material 5. Supplementary Table 1. The characteristics of the populations between the those with missing values and those without.

Supplementary Material 6. Supplementary Table 2. Associations of AFB, and ALB with history of total CVD in women in the United States from NHANES 1999–2018.

Supplementary Material 7. Supplementary Table 3. Associations of number of pregnancies, and live births with history of total CVD in women in the United States from NHANES 1999–2018.

Supplementary Material 8. Supplementary Table 4. Associations of AFB with history of individual CVD in women in the United States from NHANES 1999–2018.

Supplementary Material 9. Supplementary Table 5. Associations of ALB with the history of individual CVD in women in the United States from NHANES 1999–2018.

Supplementary Material 10. Supplementary Table 6. Associations of number of pregnancies with history of individual CVD in women in the United States from NHANES 1999–2018.

Supplementary Material 11. Supplementary Table 7. Associations of number of live births with the history of individual CVD in women in the United States from NHANES 1999–2018.

## Data Availability

The survey data are publicly available on the Internet for data users and researchers throughout the world https://www.cdc.gov/nchs/nhanes/.
