# Supplementary figures and images for: The association of female reproductive factors with history of cardiovascular disease: a large cross-sectional study
Source: BMC Public Health. 2024 Jun 17;24:1616. doi: 10.1186/s12889-024-19130-4 (PMC11181605; doi:10.1186/s12889-024-19130-4)

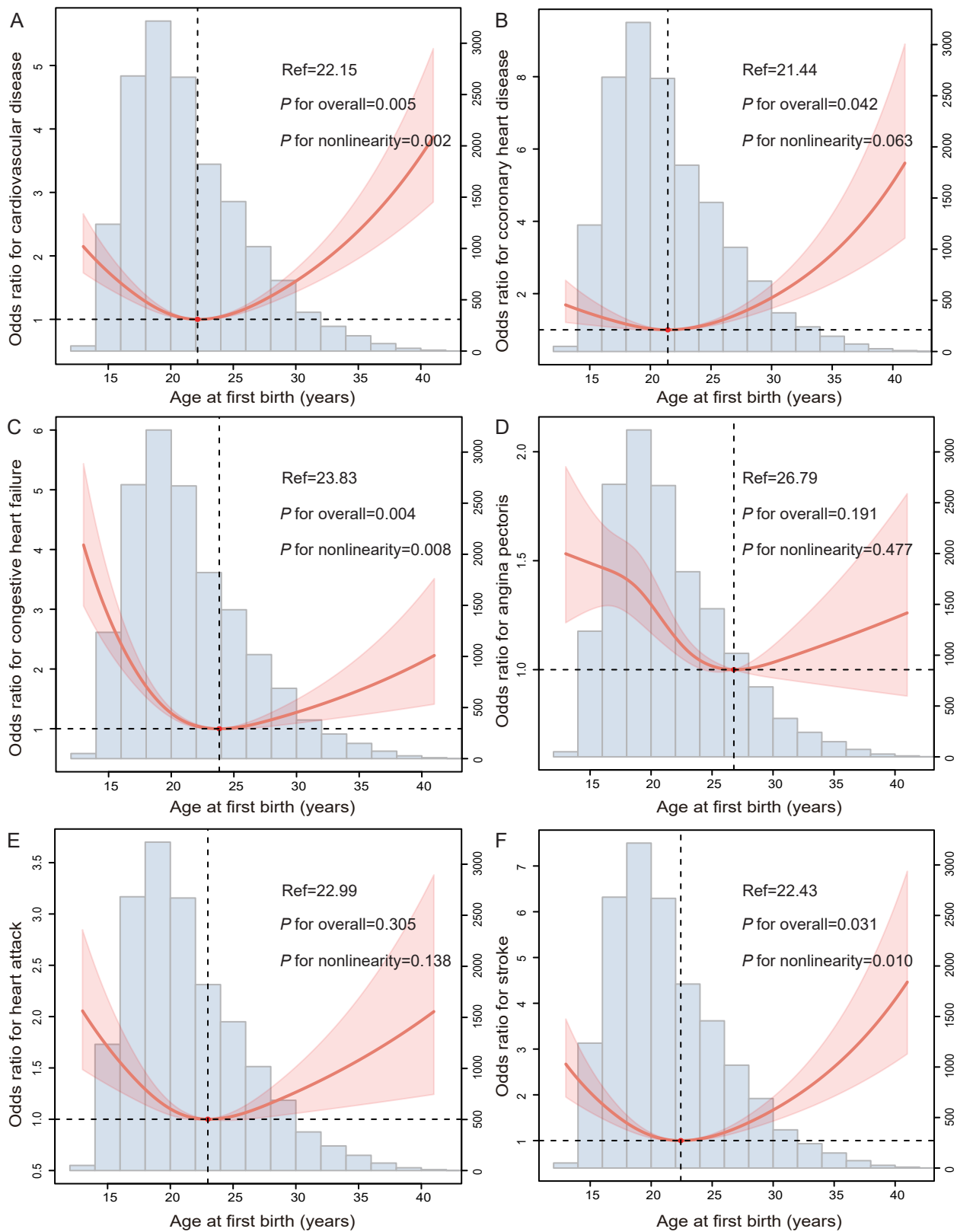

Supplement: Supplementary file 1 — Supplementary Material 1: Supplementary Figure 1. The RCS curve of the association of AFB with (A) total CVD, (B) CHD, (C) CHF, (D) angina pectoris, (E) heart attack, and (F) stroke. Abbreviation: RCS, restricted cubic spline; AFB, Age at first birth; CVD, cardiovascular disease; CHD, coronary heart disease; CHF, congestive heart failure. [file 12889_2024_19130_MOESM1_ESM.pdf]

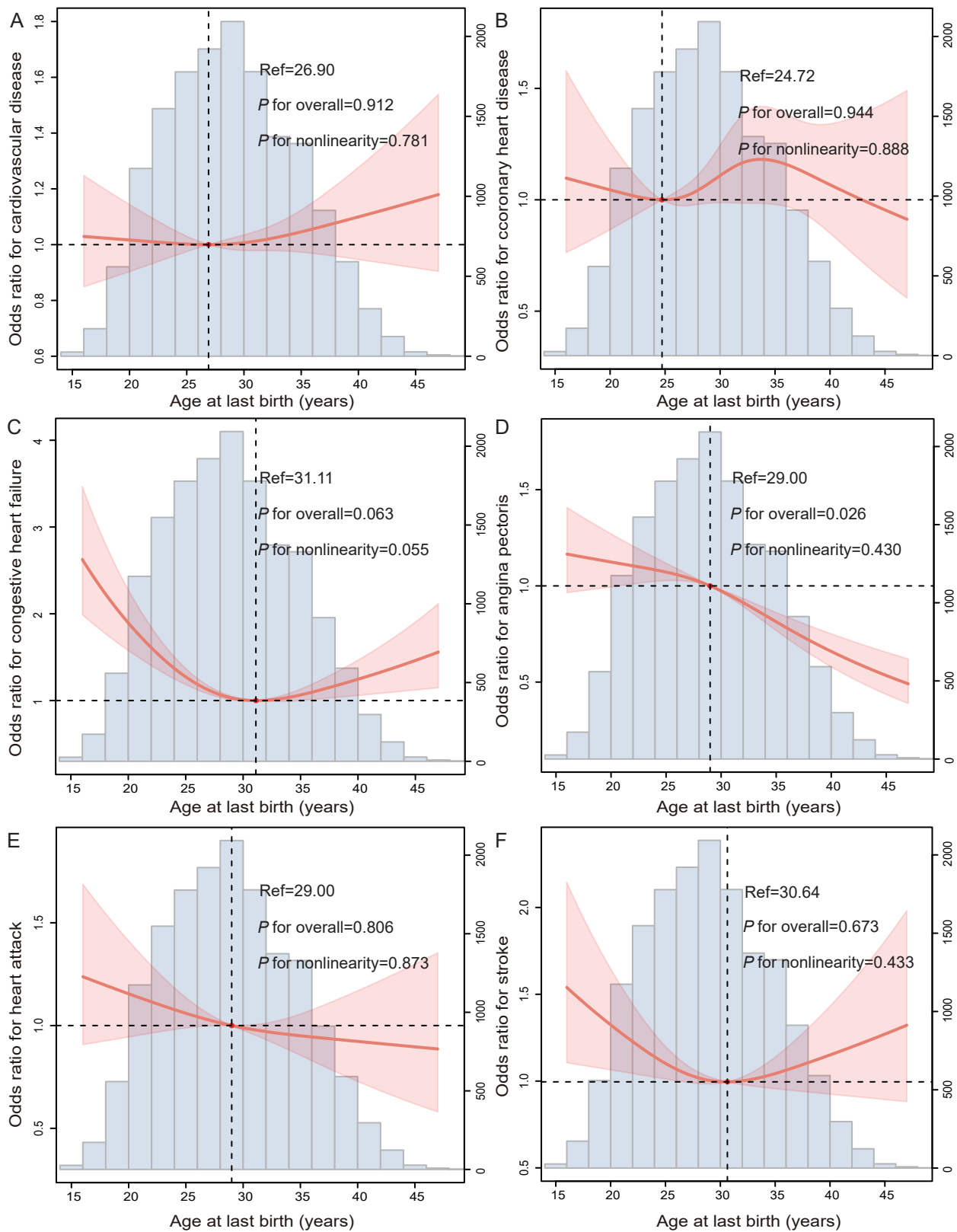

Supplement: Supplementary file 2 — Supplementary Material 2: Supplementary Figure 2. The RCS curve of the association of ALB with (A) total CVD, (B) CHD, (C) CHF, (D) angina pectoris, (E) heart attack, and (F) stroke. Abbreviation: RCS, restricted cubic spline; ALB, Age at last birth; CVD, cardiovascular disease; CHD, coronary heart disease; CHF, congestive heart failure. [file 12889_2024_19130_MOESM2_ESM.pdf]

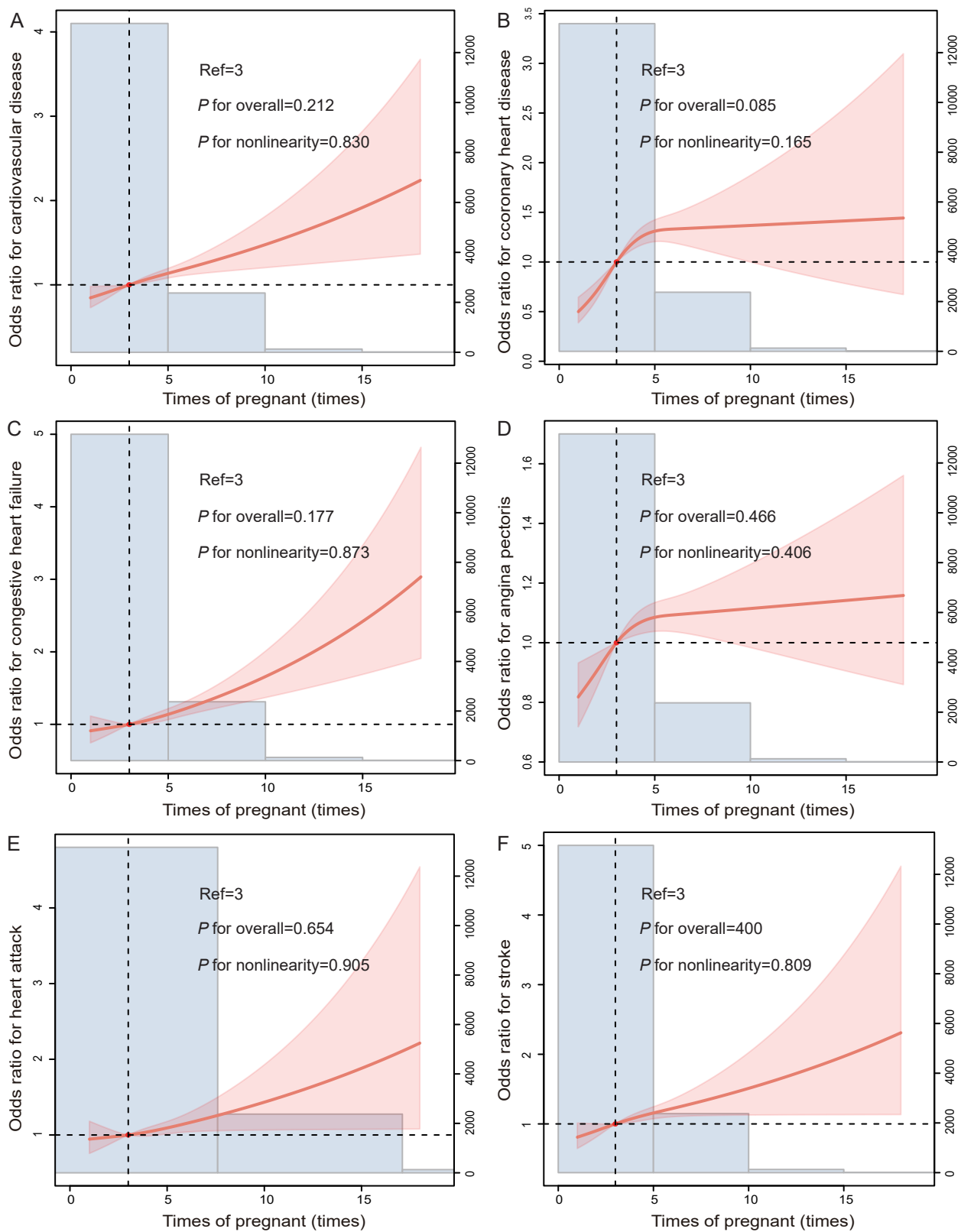

Supplement: Supplementary file 3 — Supplementary Material 3: Supplementary Figure 3. The RCS curve of the association of number of pregnancies with (A) total CVD, (B) CHD, (C) CHF, (D) angina pectoris, (E) heart attack, and (F) stroke. Abbreviation: RCS, restricted cubic spline; CVD, cardiovascular disease; CHD, coronary heart disease; CHF, congestive heart failure. [file 12889_2024_19130_MOESM3_ESM.pdf]

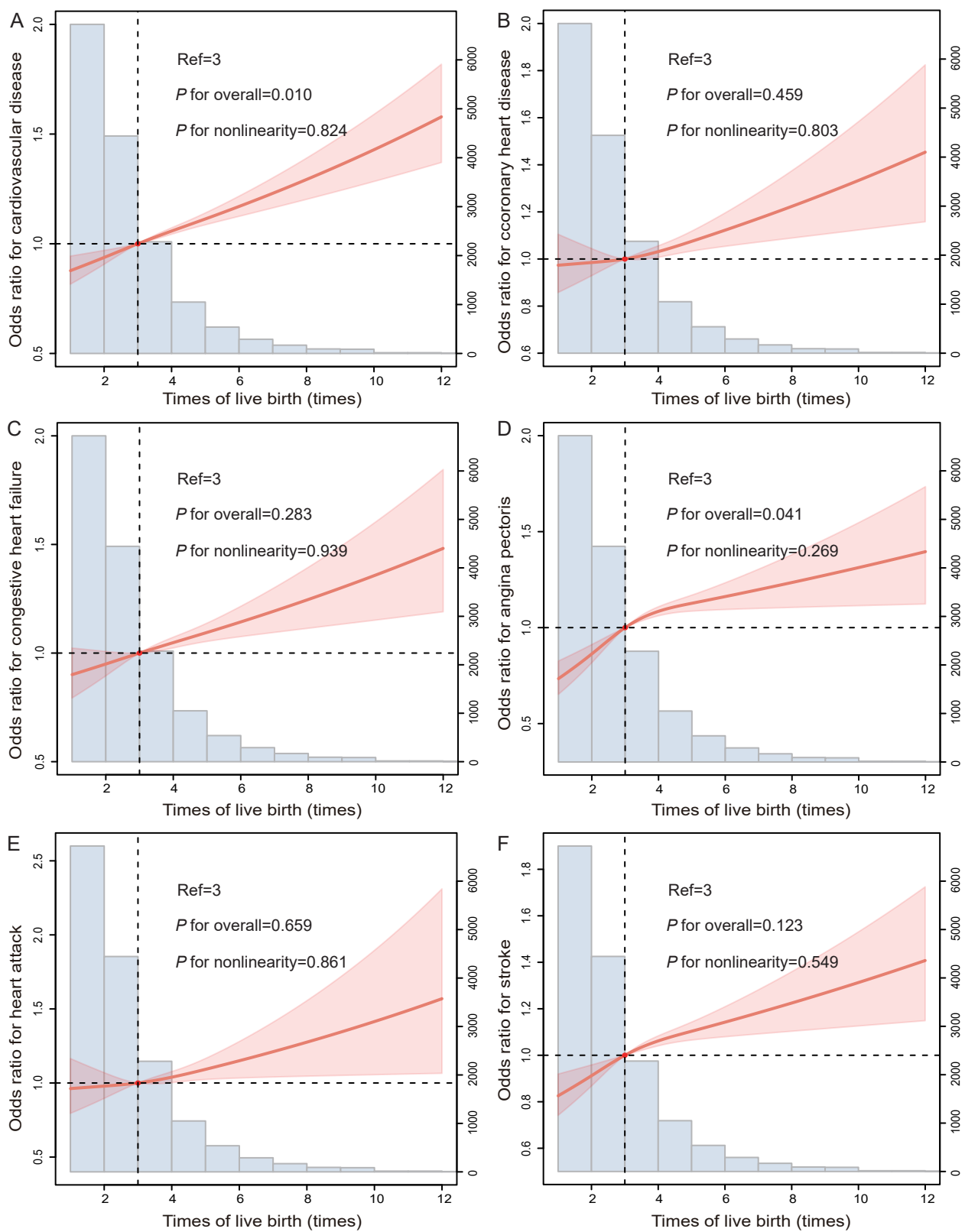

Supplement: Supplementary file 4 — Supplementary Material 4: Supplementary Figure 4. The RCS curve of the association of number of live births with (A) total CVD, (B) CHD, (C) CHF, (D) angina pectoris, (E) heart attack, and (F) stroke. Abbreviation: RCS, restricted cubic spline; CVD, cardiovascular disease; CHD, coronary heart disease; CHF, congestive heart failure. [file 12889_2024_19130_MOESM4_ESM.pdf]
