# Supplementary material for: The association of female reproductive factors with history of cardiovascular disease: a large cross-sectional study
Source: BMC Public Health. 2024 Jun 17;24:1616. doi: 10.1186/s12889-024-19130-4 (PMC11181605; doi:10.1186/s12889-024-19130-4)
Supplement: Supplementary file 5 — Supplementary Material 5. Supplementary Table 1. The characteristics of the populations between the those with missing values and those without. [file 12889_2024_19130_MOESM5_ESM.docx]

| **Supplementary Table 1**. The characteristics of the populations between the those with missing values and those without | | | | |
| --- | --- | --- | --- | --- |
| Variables | Overall (n=35,445) | Women without  missing values (n=15,715) | Women with  missing values (n=19,730) | *P*-value |
| Age, years | 41.37 ± 21.51 | 53.40 ± 16.61 | 31.79 ± 20.12 | <0.001 |
| Race, n (%) |  |  |  | <0.001 |
| Mexican American | 7213 (20.3%) | 3068 (8.7%) | 4145 (21.0%) |  |
| Other Hispanic | 2992 (8.4%) | 1406 (4.0%) | 1586 (4.4%) |  |
| Non-Hispanic Black | 8122 (22.9%) | 3217 (9.1%) | 4905 (13.8%) |  |
| Non-Hispanic White | 13907 (39.2%) | 6842 (19.3%) | 7065 (19.9%) |  |
| Other race | 3211 (9.1%) | 1182 (3.3%) | 2029 (5.7%) |  |
| Family PIR |  |  |  | <0.001 |
| < 1.3 | 12781 (36.1%) | 5455 (15.4%) | 7326 (20.7%) |  |
| ≥1.3 | 22664 (63.9%) | 10260 (28.9%) | 12404 (35.0%) |  |
| Education level, n (%) |  |  |  | <0.001 |
| Less than high school | 13951 (39.4%) | 4699 (13.3%) | 9252 (26.1%) |  |
| High school | 3268 (9.2%) | 1527 (4.3%) | 1741 (4.9%) |  |
| More than high school | 18226 (51.4%) | 9489 (26.8%) | 8737 (24.6%) |  |
| Marital status, n (%) |  |  |  | <0.001 |
| Having a partner | 15788 (44.5%) | 9346 (26.4%) | 6442 (18.2%) |  |
| No partner | 7812 (22.0%) | 5227 (14.7%) | 2585 (7.3%) |  |
| Unmarried | 11845 (33.4%) | 1142 (3.2%) | 10703 (30.2%) |  |
| Hypertension, n (%) |  |  |  | <0.001 |
| No | 24020 (67.8%) | 8211 (23.2%) | 15809 (44.6%) |  |
| Yes | 11425 (32.2%) | 7504 (21.2%) | 3921 (11.1%) |  |
| DM, n (%) |  |  |  | <0.001 |
| No | 30847 (87.0%) | 12718 (35.9%) | 18129 (51.1%) |  |
| Yes | 4598 (13.0%) | 2997 (8.5%) | 1601 (4.5%) |  |
| Smoker, n (%) |  |  |  | <0.001 |
| No | 23418 (66.1%) | 9823 (27.7%) | 13595 (38.4%) |  |
| Former | 5581 (15.7%) | 3154 (8.9%) | 2427 (6.8%) |  |
| Now | 6446 (18.2%) | 2738 (7.7%) | 3708 (10.5%) |  |
| Alcohol user, n (%) |  |  |  | <0.001 |
| No | 8963 (25.3%) | 3544 (10.0%) | 5419 (15.3%) |  |
| Former | 5053 (14.3%) | 2964 (8.4%) | 2089 (5.9%) |  |
| Mild | 8331 (23.5%) | 4452 (12.6%) | 3879 (10.9%) |  |
| Moderate | 5803 (16.4%) | 2656 (7.5%) | 3147 (8.9%) |  |
| Heavy | 7295 (20.6%) | 2099 (5.9%) | 5196 (14.7%) |  |
| Menopause status, n (%) |  |  |  | <0.001 |
| No | 23484 (66.3%) | 6779 (19.1%) | 16705 (47.1%) |  |
| Yes | 11961 (33.7%) | 8936 (25.2%) | 3025 (8.5%) |  |
| Oral contraceptive use, n (%) |  |  |  | <0.001 |
| No | 16840 (47.5%) | 5391 (15.2%) | 11449 (32.3%) |  |
| Yes | 18605 (52.5%) | 10324 (29.1%) | 8281 (23.4%) |  |
| Use female hormones, n (%) |  |  |  | <0.001 |
| No | 30214 (85.2%) | 11985 (33.8%) | 18229 (51.4%) |  |
| Yes | 5231 (14.8%) | 3730 (10.5%) | 1501 (4.2%) |  |
| Had a hysterectomy, n (%) |  |  |  | <0.001 |
| No | 29504 (83.2%) | 11423 (32.2%) | 18081 (51.0%) |  |
| Yes | 5941 (16.8%) | 4292 (12.1%) | 1649 (4.7%) |  |
| Both ovaries removed, n (%) |  |  |  | <0.001 |
| No | 31727 (89.5%) | 13087 (36.9%) | 18640 (52.6%) |  |
| Yes | 3718 (10.5%) | 2628 (16.7%) | 1090 (3.1%) |  |
| Work activity, n (%) |  |  |  | <0.001 |
| No | 21272 (60.0%) | 9223 (26.0%) | 12049 (34.0%) |  |
| Yes | 14173 (40.0%) | 6492 (18.3%) | 7681 (21.7%) |  |
| Recreational activity, n (%) |  |  |  | <0.001 |
| No | 21190 (59.8%) | 10557 (29.8%) | 10633 (30.0%) |  |
| Yes | 14255 (40.2%) | 5158 (14.6%) | 9097 (25.7%) |  |
| BMI, kg/m^2^ | 28.24 ± 7.52 | 29.86 ± 7.20 | 26.95 ± 7.51 | <0.001 |
| Waist circumference, cm | 93.56 ± 17.35 | 98.46 ± 15.82 | 89.66 ± 17.54 | <0.001 |
| Hb, g/dL | 13.25 ± 1.24 | 13.29 ± 1.30 | 13.22 ± 1.18 | <0.001 |
| Mean energy | 1777.62 ± 689.19 | 1722.32 ± 653.60 | 1821.66 ± 713.24 | <0.001 |
| intake (kcal/day) |  |  |  |  |
| TC, mg/dL | 190.61 ± 42.82 | 201.43 ± 42.19 | 182.00 ± 41.35 | 0.087 |
| TG, mg/dL | 115.95 ± 87.90 | 130.85 ± 99.20 | 104.08 ± 75.65 | <0.001 |
| HDL-C, mg/dL | 56.94 ± 15.74 | 57.25 ± 16.30 | 56.69 ± 15.28 | <0.001 |
| BUN, mg/dL | 12.13 ± 5.62 | 13.40 ± 6.11 | 11.12 ± 4.97 | <0.001 |
| UA, mg/dL | 4.72 ± 1.27 | 1.89 ± 1.34 | 4.57 ± 1.18 | <0.001 |
| Scr, mg/dL | 0.74 ± 0.35 | 0.78 ± 0.39 | 0.71 ± 0.31 | <0.001 |
| eGFR, ml/min/1.73m^2^ | 104.90 ± 28.87 | 92.06 ± 24.67 | 115.13 ± 27.87 | <0.001 |
| Age at menarche, years | 12.43 ± 2.12 | 12.78 ± 1.76 | 12.15 ± 2.34 | <0.001 |
| Age at menopause, years | 35.52 ± 13.14 | 42.24 ± 9.37 | 30.17 ± 13.24 | <0.001 |
| Fertile lifespan, years | 23.09 ± 12.80 | 29.46 ± 9.41 | 18.02 ± 12.87 | <0.001 |

Abbreviations: Data are presented as mean ± SD or n (%). CVD, cardiovascular disease; DM, diabetes mellitus; BMI, body mass index; Hb, hemoglobin; BUN, blood urea nitrogen; UA, uric acid; Scr, serum creatinine; TC, total cholesterol; TG, triglycerides; HDL-cholesterol, high density lipoprotein-cholesterol; eGFR, estimated glomerular filtration rate.
