# Supplementary material for: The association of female reproductive factors with history of cardiovascular disease: a large cross-sectional study
Source: BMC Public Health. 2024 Jun 17;24:1616. doi: 10.1186/s12889-024-19130-4 (PMC11181605; doi:10.1186/s12889-024-19130-4)
Supplement: Supplementary file 6 — Supplementary Material 6. Supplementary Table 2. Associations of AFB, and ALB with history of total CVD in women in the United States from NHANES 1999–2018. [file 12889_2024_19130_MOESM6_ESM.docx]

| **Supplementary Table 2.** Associations of AFB, and ALB with history of total CVD in women in the United States from NHANES 1999–2018 | | | | | |  |
| --- | --- | --- | --- | --- | --- | --- |
|  | Model 1 |  | Model 2 |  | Model 3 |  |
|  | OR (95%CI) | P for trend (Adjusted) | OR (95%CI) | *P* for trend (Adjusted) | OR (95%CI) | *P* for trend (Adjusted) |
| AFB |  | 0.344 (0.688) | | 0.689 (0.999) |  | 0.155 (0.310) |
| 25-27 (2046) | 1.00 |  | 1.00 |  | 1.00 |  |
| < 25 (11673) | 1.65 (1.38, 1.96) *** | | 1.33 (1.10, 1.61) ** |  | 1.08 (0.75, 1.46) |  |
| 28-34 (1736) | 1.19 (0.87, 1.37) | | 1.15 (0.87, 1.51) | | 1.47 (0.83, 2.60) |  |
| > 34 (260) | 1.52 (1.07, 1.98) * | | 1.65 (0.93, 2.20) | | 1.74 (0.60, 5.02) |  |
| ALB |  | 0.286 (0.572) | | 0.490 (0.980) |  | 0.581 (0.999) |
| 25-29 (4708) | 1.00 |  | 1.00 |  | 1.00 |  |
| < 25 (3479) | 1.00 (0.87, 1.16) | | 1.06 (0.92, 1.22) |  | 1.03 (0.79, 1.37) |  |
| 30-34 (4236) | 1.13 (0.90, 1.27) | | 1.07 (0.92, 1.24) | | 1.06 (0.74, 1.37) |  |
| > 34 (3292) | 1.33 (1.14, 1.55) *** | | 1.10 (0.93, 1.30) | | 1.09 (0.89, 1.41) |  |

Abbreviations: AFB, age at first birth; ALB, age at last birth; CVD, cardiovascular disease; ****P* < 0.001; OR, odd ratio; CI, confidence interval. Model 1: age and race/ethnicity. Model 2: model 1 variables plus education level, marital status, family poverty-income ratio, hypertension, diabetes mellitus, smoker, alcohol user; Model 3 was adjusted for model 2 variables plus body mass index, waist circumference, mean energy intake, hemoglobin, fast glucose, glycosylated hemoglobin, menopause status, oral contraceptive use, use female hormones, had a hysterectomy, both ovaries removed, blood urea nitrogen, uric acid, serum creatinine, estimated glomerular filtration rate, total cholesterol, triglyceride, high-density lipoprotein-cholesterol, time of live birth, time of pregnant, age at menarche, age at menopause, and fertile lifespan. Of these, 13,997 were non-CVD and 1,718 were CVD.
