# Supplementary material for: The association of female reproductive factors with history of cardiovascular disease: a large cross-sectional study
Source: BMC Public Health. 2024 Jun 17;24:1616. doi: 10.1186/s12889-024-19130-4 (PMC11181605; doi:10.1186/s12889-024-19130-4)
Supplement: Supplementary file 7 — Supplementary Material 7. Supplementary Table 3. Associations of number of pregnancies, and live births with history of total CVD in women in the United States from NHANES 1999–2018. [file 12889_2024_19130_MOESM7_ESM.docx]

| **Supplementary Table 3.** Associations of number of pregnancies, and live births with history of total CVD in women in the United States from NHANES 1999–2018 | | | | | |  |
| --- | --- | --- | --- | --- | --- | --- |
|  | Model 1 |  | Model 2 |  | Model 3 |  |
|  | OR (95%CI) | *P* for trend (Adjusted) | OR (95%CI) | *P* for trend (Adjusted) | OR (95%CI) | *P* for trend (Adjusted) |
| Number of |  | <0.001 (<0.001) | | 0.005 (0.010) |  | 0.078 (0.156) |
| pregnancies |  |  | |  |  |  |
| 0-2 (4225) | 1.00 |  | 1.00 |  | 1.00 |  |
| 3 (4122) | 1.08 (0.94, 1.25) | | 1.09 (0.93, 1.27) |  | 1.04 (0.82, 1.28) |  |
| 4 (3141) | 1.16 (0.99, 1.36) | | 1.19 (0.98, 1.41) | | 1.07 (0.85, 1.43) |  |
| >5 (4227) | 1.40 (1.21, 1.61) *** | | 1.22 (1.04, 1.44) * | | 1.20 (0.92, 1.56) |  |
| Number of |  | <0.001 (<0.001) | | 0.016 (0.032) |  | 0.647 (0.999) |
| live births |  |  | |  |  |  |
| 0-2 (6849) | 1.00 |  | 1.00 |  | 1.00 |  |
| 3 (4444) | 1.11 (0.97, 1.27) | | 1.05 (0.90, 1.22) |  | 1.02 (0.76, 1.36) |  |
| 4 (2282) | 1.32 (1.13, 1.54) *** | | 1.14 (0.96, 1.36) | | 1.04 (0.81, 1.32) |  |
| >5 (2140) | 1.48 (1.27, 1.72) *** | | 1.22 (1.03, 1.45) * | | 1.16 (0.88, 1.54) |  |

Abbreviations: CVD, cardiovascular disease; **P* < 0.05, ****P* < 0.001; OR, odd ratio; CI, confidence interval. Model 1: age and race/ethnicity. Model 2: model 1 variables plus education level, marital status, family poverty-income ratio, hypertension, diabetes mellitus, smoker, alcohol user; Model 3 was adjusted for model 2 variables plus body mass index, waist circumference, mean energy intake, hemoglobin, fast glucose, glycosylated hemoglobin, menopause status, oral contraceptive use, use female hormones, had a hysterectomy, both ovaries removed, blood urea nitrogen, uric acid, serum creatinine, estimated glomerular filtration rate, total cholesterol, triglyceride, high-density lipoprotein-cholesterol, time of live birth, time of pregnant, age at menarche, age at menopause, and fertile lifespan. Of these, 13,997 were non-CVD and 1,718 were CVD.
