# Supplementary material for: The association of female reproductive factors with history of cardiovascular disease: a large cross-sectional study
Source: BMC Public Health. 2024 Jun 17;24:1616. doi: 10.1186/s12889-024-19130-4 (PMC11181605; doi:10.1186/s12889-024-19130-4)
Supplement: Supplementary file 8 — Supplementary Material 8. Supplementary Table 4. Associations of AFB with history of individual CVD in women in the United States from NHANES 1999–2018. [file 12889_2024_19130_MOESM8_ESM.docx]

| **Supplementary Table 4.** Associations of AFB with history of individual CVD in women in the United States from NHANES 1999–2018 | | | | | |
| --- | --- | --- | --- | --- | --- |
| AFB | CHD | CHF | Angina pectoris | Heart attack | Stroke |
|  | OR (95%CI) | OR (95%CI) | OR (95%CI) | OR (95%CI) | OR (95%CI) |
| 25-27 (2046) | 1.00 | 1.00 | 1.00 | 1.00 | 1.00 |
| < 25 (11673) | 1.06 (0.61, 1.92) | 1.51 (1.05, 2.19) * | 1.48 (1.04, 2.11) * | 1.27 (0.73, 1.79) | 1.56 (0.93, 1.94) |
| 28-34 (1736) | 1.38 (0.56, 3.40) | 1.47 (0.89, 2.43) | 1.36 (0.84, 2.21) | 1.20 (0.54, 2.37) | 1.67 (0.99, 2.56) |
| > 34 (260) | 3.88 (0.98, 8.44) | 1.59 (0.94, 2.55) | 1.83 (0.95, 3.75) | 1.80 (0.37, 8.62) | 1.91 (0.91, 4.80) |
| *P* for trend (Adjusted) | 0.156 (0.312) | 0.340 (0.680) | 0.359 (0.718) | 0.846 (0.999) | 0.280 (0.560) |

Abbreviations: CVD, cardiovascular disease; AFB, age at first birth; CHD, coronary heart disease; CHF, congestive heart failure; **P* <0.05; OR, odd ratio; CI, confidence interval. Analysis was adjusted for age, race/ethnicity, education level, marital status, family poverty-income ratio, hypertension, diabetes mellitus, smoker, alcohol user, body mass index, waist circumference, mean energy intake, hemoglobin, fast glucose, glycosylated hemoglobin, menopause status, oral contraceptive use, use female hormones, had a hysterectomy, both ovaries removed, blood urea nitrogen, uric acid, serum creatinine, estimated glomerular filtration rate, total cholesterol, triglyceride, high-density lipoprotein-cholesterol, time of live birth, time of pregnant, age at menarche, age at menopause, and fertile lifespan. Of these, 15,214 women were non-CHD and 501 women were CHD; 15,215 women were non-CHF and 500 women were CHF; 15,253 women were angina pectoris and 462 women were non-angina pectoris; 15,166 were non-heart attack and 549 women were heart attack; 15,041 were non-stroke and 674 women were stroke.
