# Supplementary material for: The association of female reproductive factors with history of cardiovascular disease: a large cross-sectional study
Source: BMC Public Health. 2024 Jun 17;24:1616. doi: 10.1186/s12889-024-19130-4 (PMC11181605; doi:10.1186/s12889-024-19130-4)
Supplement: Supplementary file 9 — Supplementary Material 9. Supplementary Table 5. Associations of ALB with the history of individual CVD in women in the United States from NHANES 1999–2018. [file 12889_2024_19130_MOESM9_ESM.docx]

| **Supplementary Table 5.** Associations of ALB with the history of individual CVD in women in the United States from NHANES 1999–2018 | | | | | |
| --- | --- | --- | --- | --- | --- |
| ALB | CHD | CHF | Angina pectoris | Heart attack | Stroke |
|  | OR (95%CI) | OR (95%CI) | OR (95%CI) | OR (95%CI) | OR (95%CI) |
| 25-29 (4708) | 1.00 | 1.00 | 1.00 | 1.00 | 1.00 |
| < 25 (3479) | 1.07 (0.71, 1.68) | 1.17 (0.85, 1.61) | 1.26 (0.92, 1.71) | 1.14 (0.77, 1.54) | 1.07 (0.72, 1.44) |
| 30-34 (4236) | 1.06 (0.68, 1.55) | 1.14 (0.80, 1.37) | 1.02 (0.73, 1.40) | 0.89 (0.51, 1.55) | 1.01 (0.65, 1.50) |
| > 34 (3292) | 1.12 (0.72, 1.61) | 1.01 (0.68, 1.21) | 1.14 (0.83, 1.34) | 0.71 (0.42, 1.20) | 1.10 (0.65, 1.88) |
| *P* for trend (Adjusted) | 0.446 (0.892) | 0.125 (0.250) | 0.145 (0.290) | 0.408 (0.816) | 0.846 (0.999) |

Abbreviations: CVD, cardiovascular disease; ALB, age at last birth; CHD, coronary heart disease; CHF, congestive heart failure; OR, odd ratio; CI, confidence interval. Analysis was adjusted for age, race/ethnicity, education level, marital status, family poverty-income ratio, hypertension, diabetes mellitus, smoker, alcohol user, body mass index, waist circumference, mean energy intake, hemoglobin, fast glucose, glycosylated hemoglobin, menopause status, oral contraceptive use, use female hormones, had a hysterectomy, both ovaries removed, blood urea nitrogen, uric acid, serum creatinine, estimated glomerular filtration rate, total cholesterol, triglyceride, high-density lipoprotein-cholesterol, time of live birth, time of pregnant, age at menarche, age at menopause, and fertile lifespan. Of these, 15,214 women were non-CHD and 501 women were CHD; 15,215 women were non-CHF and 500 women were CHF; 15,253 women were angina pectoris and 462 women were non-angina pectoris; 15,166 were non-heart attack and 549 women were heart attack; 15,041 were non-stroke and 674 women were stroke.
