# Supplementary material for: The association of female reproductive factors with history of cardiovascular disease: a large cross-sectional study
Source: BMC Public Health. 2024 Jun 17;24:1616. doi: 10.1186/s12889-024-19130-4 (PMC11181605; doi:10.1186/s12889-024-19130-4)
Supplement: Supplementary file 10 — Supplementary Material 10. Supplementary Table 6. Associations of number of pregnancies with history of individual CVD in women in the United States from NHANES 1999–2018. [file 12889_2024_19130_MOESM10_ESM.docx]

| **Supplementary Table 6.** Associations of number of pregnancies with history of individual CVD in women in the United States from NHANES 1999–2018 | | | | | |
| --- | --- | --- | --- | --- | --- |
| Number of pregnancies | CHD | CHF | Angina pectoris | Heart attack | Stroke |
|  | OR (95%CI) | OR (95%CI) | OR (95%CI) | OR (95%CI) | OR (95%CI) |
| 0-2 (4225) | 1.00 | 1.00 | 1.00 | 1.00 | 1.00 |
| 3 (4122) | 1.69 (0.85, 3.36) | 1.11 (0.81, 1.52) | 1.05 (0.77, 1.40) | 1.17 (0.76, 1.89) | 1.17 (0.73, 1.96) |
| 4 (3141) | 2.31 (1.24, 4.29) * | 1.13 (0.84, 1.53) | 1.10 (0.80, 1.51) | 1.31 (0.75, 2.31) | 1.20 (0.70, 2.05) |
| >5 (4227) | 2.46 (1.33, 4.55) ** | 1.31 (1.00, 1.73) * | 1.43 (1.08, 1.89) | 1.35 (0.80, 2.30) | 1.56 (0.94, 2.61) |
| *P* for trend (Adjusted) | 0.021 (0.042) | 0.062 (0.124) | 0.006 (0.012) | 0.781 (0.999) | 0.123 (0.246) |

Abbreviations: CVD, cardiovascular disease; AFB, age at first birth; CHD, coronary heart disease; CHF, congestive heart failure; **P* <0.05; ***P* <0.01; OR, odd ratio; CI, confidence interval. Analysis was adjusted for age, race/ethnicity, education level, marital status, family poverty-income ratio, hypertension, diabetes mellitus, smoker, alcohol user, body mass index, waist circumference, systolic blood pressure, diastolic blood pressure, mean energy intake, hemoglobin, fast glucose, glycosylated hemoglobin, menopause status, oral contraceptive use, use female hormones, had a hysterectomy, both ovaries removed, blood urea nitrogen, uric acid, serum creatinine, estimated glomerular filtration rate, total cholesterol, triglyceride, high-density lipoprotein-cholesterol, time of live birth, time of pregnant, age at menarche, age at menopause, and fertile lifespan. Of these, 15,214 women were non-CHD and 501 women were CHD; 15,215 women were non-CHF and 500 women were CHF; 15,253 women were angina pectoris and 462 women were non-angina pectoris; 15,166 were non-heart attack and 549 women were heart attack; 15,041 were non-stroke and 674 women were stroke.
