# Supplementary material for: The association of female reproductive factors with history of cardiovascular disease: a large cross-sectional study
Source: BMC Public Health. 2024 Jun 17;24:1616. doi: 10.1186/s12889-024-19130-4 (PMC11181605; doi:10.1186/s12889-024-19130-4)
Supplement: Supplementary file 11 — Supplementary Material 11. Supplementary Table 7. Associations of number of live births with the history of individual CVD in women in the United States from NHANES 1999–2018. [file 12889_2024_19130_MOESM11_ESM.docx]

| **Supplementary Table 7.** Associations of number of live births with the history of individual CVD in women in the United States from NHANES 1999–2018 | | | | | |
| --- | --- | --- | --- | --- | --- |
| Number of live births | CHD | CHF | Angina pectoris | Heart Attack | Stroke |
|  | OR (95%CI) | OR (95%CI) | OR (95%CI) | OR (95%CI) | OR (95%CI) |
| 0-2 (6849) | 1.00 | 1.00 | 1.00 | 1.00 | 1.00 |
| 3 (4444) | 1.03 (0.57, 1.88) | 1.05 (0.75, 1.22) | 1.12 (0.87, 1.41) | 1.07 (0.68, 1.46) | 1.13 (0.71, 1.59) |
| 4 (2282) | 1.24 (0.63, 2.16) | 1.23 (0.86, 1.59) | 1.27 (0.94, 1.55) | 1.22 (0.94, 1.54) | 1.37 (0.94, 1.81) |
| >5 (2140) | 1.45 (0.70, 2.38) | 1.53 (0.93, 2.03) | 1.42 (0.98, 1.84) | 1.39 (0.98, 1.66) | 1.53 (0.98, 2.09) |
| *P* for trend (Adjusted) | 0.732 (0.999) | 0.120 (0.240) | 0.188 (0.376) | 0.105 (0.210) | 0.603 (0.999) |

Abbreviations: CVD, cardiovascular disease; ALB, age at last birth; CHD, coronary heart disease; CHF, congestive heart failure; OR, odd ratio; CI, confidence interval. Analysis was adjusted for age, race/ethnicity, education level, marital status, family poverty-income ratio, hypertension, diabetes mellitus, smoker, alcohol user, body mass index, waist circumference, mean energy intake, hemoglobin, fast glucose, glycosylated hemoglobin, menopause status, oral contraceptive use, use female hormones, had a hysterectomy, both ovaries removed, blood urea nitrogen, uric acid, serum creatinine, estimated glomerular filtration rate, total cholesterol, triglyceride, high-density lipoprotein-cholesterol, time of live birth, time of pregnant, age at menarche, age at menopause, and fertile lifespan. Of these, 15,214 women were non-CHD and 501 women were CHD; 15,215 women were non-CHF and 500 women were CHF; 15,253 women were angina pectoris and 462 women were non-angina pectoris; 15,166 were non-heart attack and 549 women were heart attack; 15,041 were non-stroke and 674 women were stroke.
